# Emerging Opportunities of Radiotherapy Combined With Immunotherapy in the Era of Breast Cancer Heterogeneity

**DOI:** 10.3389/fonc.2018.00609

**Published:** 2018-12-12

**Authors:** Pelagia G. Tsoutsou, Khalil Zaman, Silvia Martin Lluesma, Laurene Cagnon, Lana Kandalaft, Marie-Catherine Vozenin

**Affiliations:** ^1^Division of Oncology, Radio-oncology Department, Vaudois University Hospital Centre (CHUV), Lausanne, Switzerland; ^2^Radio-Oncology Research Laboratory, Vaudois University Hospital Centre (CHUV), Epalinges, Switzerland; ^3^Radiation Oncology Department, Hôpital Neuchâtelois, La Chaux-de-Fonds, Switzerland; ^4^Department of Oncology, Breast Center, Vaudois University Hospital Centre (CHUV), Lausanne, Switzerland; ^5^Department of Oncology, Center of Experimental Therapeutics, Ludwig Center for Cancer Research, University of Lausanne, Lausanne, Switzerland

**Keywords:** breast cancer, subtypes, immunotherapy, radiotherapy, TILs

## Abstract

The association of radiotherapy and immunotherapy has recently emerged as an exciting combination that might improve outcomes in many solid tumor settings. In the context of breast cancer, this opportunity is promising and under investigation. Given the heterogeneity of breast cancer, it might be meaningful to study the association of radiotherapy and immunotherapy distinctly among the various breast cancer subtypes. The use of biomarkers, such as tumor infiltrating lymphocytes, which are also associated to breast cancer heterogeneity, might provide an opportunity for tailored studies. This review highlights current knowledge of the association of radiotherapy and immunotherapy in the setting of breast cancer and attempts to highlight the therapeutic opportunities among breast cancer heterogeneity.

## Introduction

Breast cancer (BC) is the most frequently diagnosed cancer and leading cause of cancer death among females worldwide. BC survival is closely related to cancer biology and disease stage, in a disease setting that presents a tremendous heterogeneity in terms of natural history (1). Historically, Halsted proposed that BC represents a local disease that progressively spreads to adjacent tissues through lymphatics (2); Fisher then underlined the systemic component of the disease (3, 4) and finally Hellman suggested that BC is heterogeneous, varying from a solely local disease throughout its whole course vs. systemic disease at presentation (5, 6). Local and systemic treatments address therapeutically these two BC elements. The current challenge becomes the introduction of emerging therapies, such as immunotherapy, in association to established modalities, such as radiotherapy (RT), chemotherapy or targeted treatments in order to improve oncological outcomes. This review will examine existing data highlighting potential combinations of immunotherapy and RT in the setting of BC. The possible implications of BC heterogeneity on RT-immunotherapy combinations will also be discussed.

## Biological Considerations of BC

The molecular biological basis of BC heterogeneity was poorly understood until 2000 when Perou and colleagues brought into light at least four distinct BC subtypes (7). Tumors were classified based on their gene expression and the fact that variations of their transcriptional program were implicated in their diversity. A classification system based upon the gene expression profile of a tumor was thus developed, with selection of particular gene groups (intrinsic gene subset) constantly present in the same tumor and distinct among different tumors. Others have subsequently further dissected BC heterogeneity (8) providing tailored therapeutic targets to distinct subsets of BC.

Nowadays, BC is no longer considered a unique disease, but the clinical manifestation of several. The classification of 4 subtypes, associated to distinct clinical behaviors and natural histories of BC, has permitted refining systemic therapy in order to improve patient's outcome and minimize toxicities (9–11). Despite this, a complete image of the biological heterogeneity of BC in regards to molecular alterations, sensitivity to treatment and cellular composition, is still lacking. In clinical practice, most BC tumors are classified as Luminal A or B, HER-2 positive, or triple negative (TN) based on pathological parameters in immunohistochemistry, such as hormone receptor status, HER-2 status, grade, and proliferation index (Ki-67), that have been well defined (12), although they do not perfectly correspond to the molecular subtypes defined by Perou (13). Luminal A is the most favorable subtype in terms of prognosis and endocrine sensitivity (14), while triple negative remains a subtype with highly aggressive biology, associated with an increased risk of locoregional recurrence (15) and systemic failure (16).

## Established Treatment Options in BC

Surgery is an important treatment modality for non-metastatic disease of every stage. Neo/adjuvant systemic treatments, namely chemotherapy, endocrine therapy, and targeted treatments, have been been combined with surgery in the non-metastatic setting given their ability to reduce the risk of systemic and local recurrence (17). Systemic treatments have been the cornerstone in the metastatic setting and have been providing overall survival gains within the last decades (18). Radiotherapy is a local/locoregional treatment with the potential to sterilize residual microscopic disease in BC. It is often indicated after mastectomy, when prognostic factors imply an increased risk of locoregional recurrence (LR), and systematically after breast conserving surgery, permitting equal locoregional control rates to mastectomy and conferring an overall survival benefit (19).

The indication of a systemic treatment is now refined by the use of surrogates, which permit the selection of patients at highest risk for relapse, and thus in need for an additional “preventive” treatment (prognostic factors), as well as the patients with enhanced probabilities of response to a given treatment (predictive factors, a concept known as treatment personalization. BC is perhaps the paradigm of personalized therapy, since data accumulation, due to its high incidence, has permitted unprecedented insight into disease heterogeneity.

## Cancer Immunotherapy

Immunotherapy is an emerging modality in cancer treatment. The basic principle for introducing immunotherapy in cancer treatment is that although tumors are finally poorly immunogenic entities, which, according to the “immunosurveillance hypothesis,” escape immune detection (20), they present as initially immunogenic and are eliminated by the immune system. Natural selection results in the persistence of the less immunogenic clones, through the expression of immunosuppressive cytokines and growth factors (21), though a procedure known as “immunoediting,” which progressively enriches tumor microenvironment by immunosuppressive cell populations, such as T_reg_ (CD4^+^CD25^+^regulatory T cells) and hijacked plasmacytoid dendritic cells (pDCs) (21, 22).

The central idea of cancer immunotherapy is to identify tumor specific antigens, not present in essential normal tissues (so that autoimmune phenomena are avoided), which could induce a tumor-specific immune response and promote adaptive immunity to fight back the tumor within a given cancer patient. Immunotherapies comprise active, passive or immunomodulatory strategies, although some of them overlap (23). Vaccines or adoptive-cell therapies with autologous T-cells are active strategies aiming to increase the ability of the patients' own immune system to mount an immune response against the patient's tumor (24).

**Figure 1:**
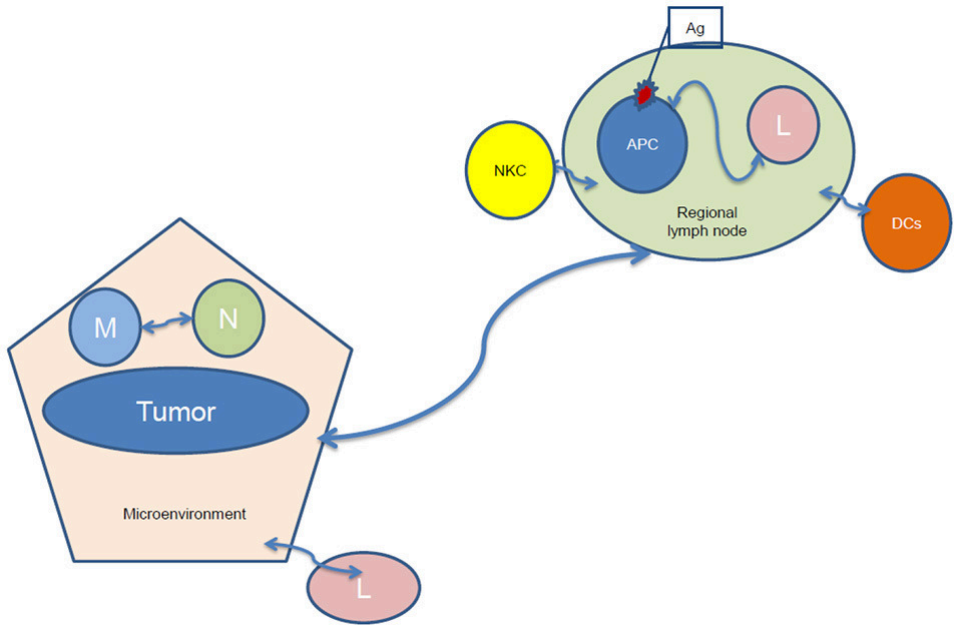
Tumor-regional lymph node communication after irradiation. NKC, natural killer cells; Ag, antigen; APC, antigen-presenting cells; L, lymphocytes; DCs, dendritic cells; M, macrophages; N, Neutrophil.

Truly tumor-specific antigens, “tumor-associated antigens” (TAAs) are rare (25), but still can be detected within the tumor and in the peripheral blood of patients with specific tumors (26). In order to identify and clone TAAs, tumor infiltrating lymphocytes (TILs) have been isolated, and emerge as a therapeutic tool (27). Both antigen presentation and lymphocyte activation depend on the tumor microenvironment (28), while the interaction of lymphocytes with antigen-presenting cells occurs in regional lymph nodes, where dendritic cells (DCs, which are professional Ag-presenting cells) migrate during their maturation process (Figure 1). DCs present antigens to lymphocytes, activating them to identify, target and destroy tumor cells (29). The microenvironment can induce tumor-suppressing and promoting pathways, including the secretion of cytokines and growth factors by stromal cells and promoting macrophage polarization, angiogenic switch and immune suppression or evasion by affecting *in situ* immune cells of myeloid and lymphoid lineage (30).

## Tumor-infiltrating lymphocytes (TILs): A biomarker of BC immunogenicity

### TILs as a Biomarker

TILs infiltrating BC are tumor specific T cells chronically exposed to tumor associated antigens (TAAs) (31). Whereas normal breast tissue does not contain large quantities of immune cells (32), TILs infiltration can be observed in specific subtypes of BC (Table 1). Notably, TILs are mainly present in TNBC and HER-2 positive BC, in which subtypes their increased number has a positive prognostic impact. Increased TILs infiltration has also been correlated to better overall prognosis and response to neo-/adjuvant chemotherapy (34) and recently, TILs have been proven an independent prognostic factor for disease-free survival (DFS) and overall survival (OS) in TNBC (34). The first studies of TILs have been published in a pivotal study published in 1992, in which the predictive value of TILs for axillary lymph-node status, tumor diameter and histological and morphometric variables has been reported in 489 BC patients after 10-year follow-up (35). More importantly, they correlated to recurrence-free survival and BC-specific survival in rapidly proliferating, axillary lymph-node negative disease (36).

**Table 1:** The median percentage of stromal tissue TILs within the various BC subtypes (33).

**BC subtype**	**Median percentage of stromal tissue TILs (%)**	**Minimum percentage of stromal tissue TILs (%)**	**Maximum percentage of stromal tissue TILs (%)**
ER- positive/HER-2 negative	10	1	75
HER-2 positive	15	0.5	80
TN	20	2.5	75

In the last five years, TILs have been evaluated in about 16,000 patients, thus highlighting the growing interest in this biomarker (35–39). Loi et al. have shown in 2,009 patients participating in the Breast International Group (BIG) 02-98 trial (a phase III trial in the adjuvant setting of early BC), that TILs are an important prognostic biomarker, but in TN patients only (33). In that study, after calculation of tumor lymphocyte infiltration (defined as the percentage of mononuclear cells within the epithelium of the invasive tumor nests) and stromal lymphocyte infiltration (defined as the percentage of infiltrating lymphocytes into the stroma), a lymphocyte predominant phenotype (LPBC) was defined as >50% infiltration of either tumoral or stromal TILs (sTILs).

As expected, TILs were higher in TN and HER-2 positive subgroups given they are highly proliferative tumors. TILs were independent predictors of DFS and OS in the TN subgroup of patients only. Importantly, for every 10% increase in TILs there was a 15–17% (stromal vs. intratumoral TILs) decrease in the risk of recurrence and a 17–27% decrease in the risk of death (stromal *vs* intratumoral TILs). The 5-year DFS was 92 vs. 62%, and the 5-year OS was 92 vs. 71% for TN patients with a LPBC phenotype vs a non LPBC phenotype, respectively. However, it should be noted that only 27 patients had a LPBC in the TN group, while 229 patients had not. In that study, TILs were also predictive of response to taxanes within the HER-2 positive subtype only (33).

The OS and DFS benefit has recently been confirmed in a meta-analysis in TNBC, where a 15–20% gain in any recurrence or mortality was shown for every 10% TILs' increase (40). In TNBC patients, intratumoral or stromal presence of TILs has been consistently associated with a survival benefit. In a meta-analysis that was conducted to identify the prognostic value of TILs and/or TILs subsets in BC patients stratified by infiltration sites, the presence of TILs was associated to improved disease-free survival (DFS) (HR = 0.82; 95% CI, 0.76–0.88 8) and overall survival (OS) in TNBC patients; (HR = 0.79; 95% CI, 0.71–0.87). Both intratumoral and stromal TILs were associated with good prognosis, while LPBC was a surrogate of a particularly significant survival benefit (41).

It was then shown in 12,439 BC patients that the presence of CD8^+^ TILs is associated with good prognosis in HER-2 positive patients (regardless of ER positivity) also (37). In that study, a 21–28% reduction (stromal vs intratumoral TILs, respectively) in the hazard of BC-specific mortality was shown in all ER negative tumors (HER-2 positive and TN); for HER-2- and ER-positive tumors, a 27% reduction in the hazard of BC-specific mortality was shown with intratumoral CD8^+^ TILs (37). Finally, in multivariate analyses of combined data coming from two large phase III randomized adjuvant BC trials, the prognostic value of TILs in TNBC has once more been confirmed (35, 42). Moreover, it was shown that the likelihood of absence of TILs increased as the number of positive nodes increased (36).

### Molecular and Physiological Features of TILs

TILs can be detected with heamatoxylin and eosin (H&E) staining on histological slides as well as with light microcopy. Additional immunohistochemistry (IHC) helps to characterize specific lymphocyte markers (31). In BC, TILs consist mainly of heterogeneous lymphocyte populations phenotyped as CD8^+^ (cytotoxic) and CD4^+^ (T helper) T cells, as well as CD19 B cells and natural killer (NK) cells (31, 43). These cell types have different functions with a variable functional significance and impact in the context of BC. They possess cytolytic and cytokine secretion properties, as well as the property to recognize unique tumor antigens (31).

TILs are functionally important, since immunomodulatory gene activation, as well as high expression of immunological gene signatures has been detected in patients with enriched TILs (44) and associated to intrinsic tumor qualities (45). These analyses were undertaken in full-face tissue sections, consisting of the entire tumor, whereas Mahmud et al. showed a prognostic significance for **CD8**^**+**^ lymphocytes at distance of the tumor (>1cm diameter from the tumor) (44). Functionally, these studies suggested that TILs in BC have a **Th1 polarization** and express immune checkpoint molecules, such as programmed cell death-1 (**PD-1**) (46). TILs express mRNA related to genes involved in T-cell activation and T-cell checkpoint receptors, such as indoleamine 2,3-dioxygenase 1 (IDO1) and markers of T-regs (34, 47). Notably, the expression of immunosuppressive markers also increases with TILs' infiltration but should not be considered as an ineffective immunity signal (31, 34, 48). It seems indeed that the stimulus of immune recognition of breast tumors is the repertoire of tumor mutant peptides, while a variable correlation between tumor mutation burden and T-cell effector function has been recognized (31, 49). TNBC is generally known to possess an increased mutation rate while TNBC tumors accumulate mutations 13.3 times faster than luminal tumors (50). It was shown that the relapse rate after chemotherapy and radiotherapy is higher in patients with BC when they carry a TLR4 loss-of-function allele, which induces an impaired innate immune response to tumor-cell death (51).

In the meta-analysis of TNBC, a clear benefit in OS was maintained for **CD8**^**+**^ and **FOXP3**^**+**^ (regulatory) TILs (40), however, given that data on these TILs' phenotype are limited, their specific prognostic value should be considered with caution. Others have shown that **CD8**^**+**^, **CD3**^**+**^, and **CD20**^**+**^ TILs are associated to better response to neo-adjuvant chemotherapy (52). Moreover, the prognostic value of FOXP3^+^ TILs seems to be related to ER positivity: FOXP3^+^ TILs are related to improved prognosis in ER-negative tumors while they are significantly associated with poor survival in ER-positive BC (53). The expression of **PDL-1** in BC tumor cells is associated with elevated TILs and longer recurrence-free survival suggesting a functional link between TILs and tumor PD-L1 upregulation (48, 54). High intratumoral and stromal **CD3**^**+**^**, CD4**^**+**^, and **CD8**^**+**^ TILs have been also shown to be prognosticators of OS in 150 patients with BC (all subtypes represented) (54). In a meta-analysis, CD3^+^, CD8^+^ and the ratio of CD8^+^/FOXP3^+^ TILs presented the most significant positive effect on survival (hazard ratio (HR): 0.59 (confidence interval (CI) 0.43–0.78; HR:0.71 (CI:0.62–0.82 and HR:0.48, CI:0.34–0.68, respectively) (55). The relevance of local lymphoid structures to support immune activation in response to RT has also been recently suggested by our group in medullary BC (56), a type of BC infiltrated with tertiary lymphoid structures (TLS). We showed acute and transient TLS depletion after hypo-fractionated RT, followed by a restoration phase and identified possible cellular targets (i.e., Tregs) that could be selectively modulated in subsequent studies to optimize anti-tumor immune response (56).

Hormone-receptor (HR)-positive BC is less proliferative and is expected to be less immunogenic. In fact, TILs have not been shown to maintain their prognostic value in the context of HR-positive BC and might be associated with worse survival, as shown in a pooled analysis of 3,771 patients treated with neo-adjuvant therapy (57). The role of TILs seems to be unclear in this setting (58), while the surprising finding that TILS are associated with poorer prognosis has also been observed in metastatic HR-positive BC patients treated with metronomic chemotherapy (59). In fact, CD4^+^ TILs display a positive prognostic value in patients with HR-negative tumors, while FOXP3^+^/CD8^+^ TILs display a negative prognostic value in those with HR-positive tumors (60). However, it has been shown that HR-positive tumors possess lower CD8^+^ TILs, while a minority possesses high FOXP3^+^ cells (61).

### TILs, a Biomarker Beyond Prognosis

Despite the emerging importance of TILs as prognosticators and predictive factors, several points need to be addressed before their introduction into clinical practice. This has also been the case in other tumor sites, such as ovarian cancer (62). In this disease, a genetic background has been implicated in the mechanism associated with TILs infiltration (63).

Our group believes that TILs represent a biomarker beyond prognosis with major therapeutic implications in BC (64). It remains an open question if this intrinsic quality can be manipulated and a host can be “pushed” to a more favorable immunologic response within the different contexts of disease heterogeneity. This is particularly important mainly in the early BC setting, where a favorable immune response could eradicate microscopic, dormant disease that would eventually manifest as metastasis; moreover, this could also optimize local control, therefore providing an essential component of disease eradication both at the local, as well as at the regional and distant setting. According to the immunosurveillance hypothesis, poorly differentiated tumors were shown to be more antigenic and therefore stimulate a stronger immunogenic response (65). As discussed by Loi, this immunogenic response might not be sufficient to eradicate existing tumor, but might be important in preventing recurrence after surgery, as also shown in BC in the setting of preventing metastasis (42).

### Immunotherapy in BC

Vaccines have reached a relatively advanced stage of clinical evaluation in BC. Vaccines attempt to enhance tumor killing by reinforcing tumor-dependent cellular cytotoxicity, which relies mainly upon NK and CD8^+^ T-cells. In a study that has evaluated immunological effects of conventional treatment in preoperative and postoperative BC patients, as well as in healthy controls, NK cells' quantity and functional cytotoxicity, as well as T-cell functions were decreased in post-operative, post-chemo/radiotherapy patients, while cytokine counts were increased in pre-operative patients (66). This study suggests that early introduction of immunotherapy interventions might be more efficient (66). Immunotherapy merits testing in early disease stages, where tumor burden is less important and immunotherapy might be more efficient in eradicating microscopic disease. Therefore, the design of early phase window studies needs to be emphasized in order to fully unveil the potential of this modality.

Existing BC vaccines have been reviewed by Soliman (67). In these studies, patients with minimal tumor burden and those not heavily pretreated seemed to benefit most from vaccines. A hypothesis would be that antigen-specific, cytotoxic T-cells activated after vaccination are not numerically sufficient to fight against an increased tumor burden, while they might be capable of eradicating microscopic, indolent disease.

A detailed review of the role of immunotherapy in BC has suggested various strategies to introduce this modality into clinical trials (68). The concept of BC heterogeneity is important, since it has been shown that not all BC subtypes are immunogenic. The notion of immunogenic tumors, as detected by increased TILs counts, is relevant for highly proliferating tumors and notably for the HER-2 positive and TN subtypes.

### Immunotherapy in TNBC

In the paradigm of TNBC, checkpoint inhibitors have been tested and shown interesting activity profiles (69). It has been shown that 20% of TNBC express PD-L1 (70). The overall response rate (ORR) in a phase IB study of 28 metastatic TNBC patients with pembrolizumab monotherapy was 18.5% (71). Atelizolizumab has shown an ORR of 24% in 21 metastatic TNBC bearing PD-L1-positive tumors (72). Two ongoing studies of monotherapy with checkpoint inhibitors are underway (KEYNOTE-086 (NCT02447003) (phase II) and KEYNOTE-119 (NCT02555657) (randomized phase III) (69). Studies of combination of checkpoint inhibitors with chemotherapy have also been promising. A phase Ib trial of atezolizumab and nab-paclitaxel in the same setting has shown ORR of 42% (73) and two studies are currently ongoing: IMpassion130 (NCT02425891), and KEYNOTE-355 (NCT02819518) (both phase III) (69).

The TONIC trial recently showed that nivolumab in TNBC induced an ORR of 22% with a median response duration of 9 months in responders (74). Interestingly, nivolumab treatment was initiated after priming the tumor microenvironment with either irradiation or chemotherapy, resulting in a promising response rate that appeared higher than expected based on previous PD-1/PD-L1 blockade monotherapy studies in unselected TNBC. Here, the median time to response was 2.1 months, and the median response duration was 9.0 months. Median progression-free survival was 3.4 months (95% CI 2.5–3.7 months). Among patients with a complete or partial response, the 1-year overall survival rate was 83%, compared with 13% in the one patient who had stable disease.

### Immunotherapy in HER-2 Positive BC

In HER-2 positive disease, trastuzumab downregulates HER-2 signaling by blocking heterodimers; it has also been shown to activate killing of HER-2 overexpressing cells by antibody-dependent means, through activation of NK cells (75, 76). Patients with immunoglobulin fragment (IgG Fc) polymorphisms have better trastuzumab responses, through the enhancement of an immune response (77). Trastuzumab has already been combined to HER-2-specific vaccines resulting in enhanced responses (78). However, patients in the metastatic setting eventually progress and acquire resistance to these treatments. Despite trastuzumab resistance, HER-2-based vaccines, that induce polyclonal antibody responses against HER-2 have shown enhanced anti-tumor activity when administered with lapatinib in murine models. In a phase I study of a HER-2-based cancer vaccine combined with lapatinib in 12 patients with metastatic, trastuzumab-refractory, HER-2-overexpressing BC, the regimen was well tolerated and anti-HER-2-specific Ab was induced in all patients, while very satisfactory overall survival rates (1y-OS: 92%) have been observed (79).

For a comprehensive and recent overview of undergoing immunotherapy studies in BC, the reader is referred to the works of Puzstai et al. (80), Vonderheide et al. (58), and Kroemer et al. (81). Within all this evidence, it becomes apparent that immunotherapy might be an interesting strategy for maintenance or prevention of micrometastasis, while in the setting of very advanced stages with an important disease burden, it might be insufficient by itself to eradicate the disease.

### Immunotherapy in HR-Positive BC

Given the poor immunogeneicity and the ambivalent role of TILs in HR-positive BC, immunotherapy is not expected to have a major therapeutic role in this setting (69). Immunotherapy is mostly effective when sufficient neo-antigens exist, so that T-cells can be activated and BC has been shown to possess a medium mutational load (82). In fact, estrogen-receptor (ER)-positive tumors with high mutational load are associated with poorer survival (83) and immunotherapy might be an appropriate strategy in this setting, but clinical data is lacking and very few studies focused on immunotherapy in hormone-receptor positive BC are ongoing.

## RT and Immunological Effects

### Theoretical Advantages of Associating RT to Immunotherapy

The association of RT and immunotherapy has gained extensive attention in the last few years, as RT is able to modulate each parameter of the immune cycle: 1-antigen release; 2-Antigen presentation; 3-Priming and activation of T-cells; 4-T- cells trafficking to tumors; 5-T cells tumor infiltration; 6-Recognition of tumors by T cells; 7-Killing of tumor cells by T cells (25, 84–91). First, irradiation has a known tumoricidal action by provoking DNA damage through single- or double-strand DNA breaks, whose insufficient repair leads to cell death and antigen release. Therefore, intrinsic tumor radiosensitivity depends also on the immunocompetence of the host (92), and cell death caused by irradiation is also “immunogenic” (51). It has been shown that RT enhances tumor immunogenicity and increases the presence of effector immune cells to the tumor site (25, 93–95). Recently RT has been characterized as “immunomodulatory” (96) and considered as signaling “danger,” through the induction of pro-inflammatory cytokines, such as TNF-α and IL-1β (97), capable of generating an *in vivo* vaccination effect. The group of Formenti and Demaria has extensively summarized the concept of the interaction of RT with the immune system (25, 93–95).

RT-induced increased availability of tumor antigens and its immunomodulatory consequences, such as antigen capture, cell migration to the lymph nodes, polarization toward a tolerogenic or immunogenic phenotype or migration of lymphocytes into the tumor might promote tumor killing (83, 98). Combining RT with immunotherapy would therefore induce tumor cell death resulting in antigens release and promotion of DCs' maturation, enhancing the cytotoxic capacity of T cells. Therefore, a synergistic model can be conceived, where systemic effects of irradiation and immunotherapy are more effective for cancer treatment than any of both treatments administered alone (83, 98). A synergistic model of the effects of combined immunotherapy and RT is visualized in

**Figure 2:**
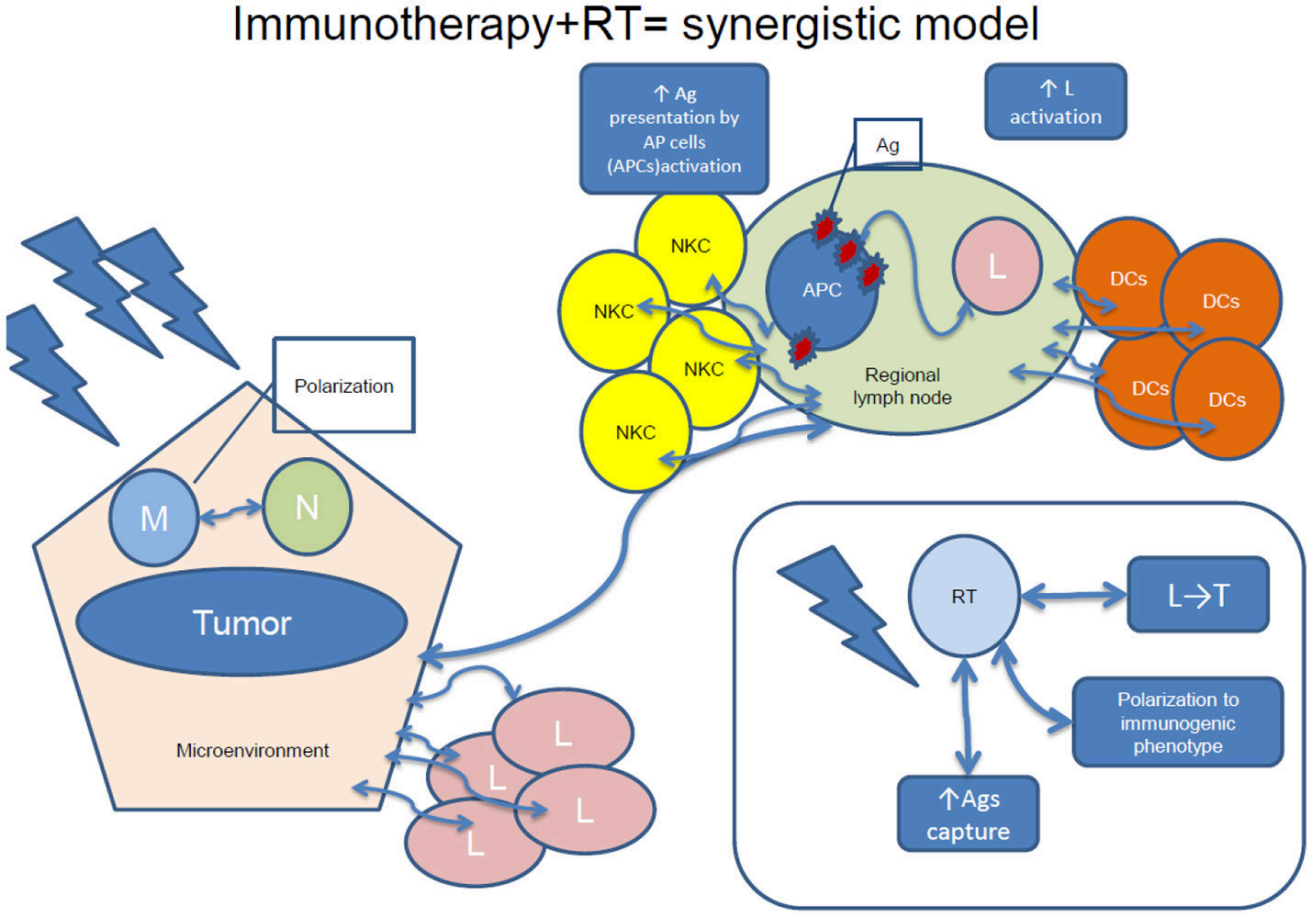
A synergistic model of immunotherapy and RT. NKC, natural killer cells; Ag, antigen; APC, antigen-presenting cells; L, lymphocytes; DCs, dendritic cells; M, macrophages; RT, radiotherapy; N, neutrophils; L, regional lymph node; T, tumor.

Figure 2.Various combinations of RT and immunotherapy have been explored, such as intratumoral (IT) or peritumoral DCs' administration, cytokines (IL-3, IL-12, TNF-α), as well as CTLA-4 blockade administration, some with promising results (22). Other combinations consist of virus, dendritic cell-based vaccines, and TLR agonists (83).

Another potential benefit of the association of RT and immunotherapy relates to the considerable evidence suggesting that RT can have inhibitory effects on tumor cells outside of the irradiation field. Formenti et al. describe four such events: (a) responses of non-irradiated tissues due to signals from irradiated cells (bystander effect), (b) effects of irradiation to the whole body (consisting of RT producing host-dependent inflammation), (c) effects of RT in tumor microenvironment, promoting phenomena outside of the treatment field, and finally (d) abscopal effects. All of these effects seem to involve the immune system (94, 95, 99–101). It remains nevertheless complex to anticipate systemic RT effects, since similar doses can provoke either pro or anti-tumorigenic effects depending upon the context. For instance, both pro- and anti-tumorigenic immune effects has been described with low doses (< 4 Gy) of irradiation (100, 102), while doses provoking cell death, such as high ablative doses of irradiation induce danger signals and activate an adaptive immune response (103) and at the same time activate immunosuppressive signals such as TGF-β1 (104).

An interplay between the primary tumor and the metastases seems to exist: in some cases, the enhancement of metastatic growth after the removal of the primary breast tumor has been observed, which was reversed when surgical removal occurred after irradiation (105–107). Although this effect remains somehow controversial in the literature and has not been extensively evaluated, it reinforces the concept of associating radiotherapy and immunotherapy in BC, a disease known par excellence to be of a strong local and systematic component (108).

### Practical Considerations of the Association

Combining the appropriate RT regimen (dose, fractionation, volume) with the appropriate immunotherapy in an appropriate schedule would therefore theoretically be locally and systemically highly effective. The real challenge today is related to our ability to determine what is the appropriate schedule in each and every tumor, capable of providing reproducible effects.

Preclinical data have shown maximum RT- immunotherapy interactions with SBRT fractions such as 6-8 Gy delivered in one to 3 fractions (83). Although immunostimulatory effects can be observed with doses as low as 0.5–0.94 Gy (109), many preclinical studies have shown that doses >7Gy increase local interferon production, which enhances antigen presentation in tumor cells (110–112). A single dose of 10 Gy has been shown to increase the efficacy of adoptive T-cell transfer *in vivo* (113). Generally, it has been shown that hypo-fractionated SBRT (single fraction of 10–24 Gy) provokes massive immunogenic release of antigens and DAMP ligands as well as stimulates TLRs on antigen-presenting cells for several days (51, 113, 114).

Notably, Dewan et al. have evaluated three different radiotherapy schemas (1 × 20 Gy, 3 × 8 Gy, and 5 × 6 Gy) in combination with a monoclonal antibody against CTLA-4 and have shown that 3 × 8 Gy was the most immunogenic combination, enriching tumors with active CD8^+^ T cells and associated to an effect on distant lesions, being either an abscopal effect or a direct effect of CTLA-4 monoclonal antibody (115). In the study by Verbugge and colleagues, immunotherapy and RT showed enhanced curative capacity of RT combined with α-CD137 and α-PD-1 antibodies in AT-3 tumors (corresponding to the triple negative subtype) with doses of 12 Gy (116), although various regimens (1 × 12 Gy, 4 × 4 Gy, or 4 × 5 Gy) were investigated. Finally, Filatenkov et al. has compared the immune modulation of 1 × 30 Gy with 10 × 3 Gy or 30 Gy + 10 × 3 Gy and has shown that 1 × 30 Gy was the most efficient regimen in terms of MDSCs decrease and CD8^+^ T-cell infiltration (117).

### Emerging Clinical Evidence and Hypotheses

The idea of combining irradiation and immunotherapy to enhance the host's immune response to tumor has been clinically evaluated in melanoma, where enhanced responses (17%) to ipilimumab were seen after irradiation of a single lesion in multimetastatic patients (118). A proof-of-principle clinical study of GM-CSF and RT managed to show the production of objective abscopal responses in oligometastatic disease, using concurrent RT of 35 Gy in 10 fractions (119). The strategy of employing high doses in small tumor volumes with tight margins seems to be the most interesting from an immunostimulatory point of view. Another recent proof-of-principle study used stereotactic body RT (SBRT) to stimulate immunity in metastatic solid tumors, associated with pembrolizumab, with doses ranging from 30 to 50 Gy in 3–5 fractions and observed that interferon-γ associated genes from tumor biopsies after SBRT were related to abscopal responses (120). Recently, Jatoi and colleagues have formulated the hypothesis that RT exerts an abscopal effect eradicating micrometastasis in the early setting of BC, which is manifested by its constant effect on distant-metastasis decrease in studies of adjuvant RT (121).

## Combining Immunotherapy and RT in BC

### Preclinical Evidence

Within BC, a preclinical study has explored the potential immunomodulatory effect of irradiation and BC (51), while the interplay between irradiation and immunotherapy has been validated in the preclinical study by Verbugge et al. (122). In mice bearing orthotopically implanted TNBC tumors, the combination of RT and immunotherapy, consisting of mAbs to CD137, CD40 and PD-1 resulted in the rejection of AT-3 and 4T1.2 tumors (122). The key innate immune cells critical to the antitumor effects of radio-immunotherapy have been those expressing CD137 and/or PD-1 and that persisted within the irradiated tumors, notably CD8^+^ and NK cells that expressed CD137. CD8^+^ T cells were essential for the therapeutic effect. In this study, anti-CD137 alone or in combination with α-CD40 treatment significantly enhanced RT-induced tumor shrinkage. It was observed that once the mice were cured of primary tumors, the growth of secondary tumors was impaired, suggesting the development of immunologic memory (122).

In another preclinical study, high-dose, ablative RT dramatically increased T-cell priming in lymphoid nodes and resulted into eradication of the primary tumor or distant metastasis also in a CD8^+^ T cell-dependent way, in mice harboring the 4T1 cell line (a TN cell line) (123). This phenomenon was greatly amplified by local immunotherapy (123). The same group had previously shown that targeting a primary breast tumor (4T1 cell line) with immunotherapy (“Ad-LIGHT, a name derived from “homologous to lymphotoxins, that shows inducible expression, and competes with herpes simplex virus glycoprotein D for herpes virus entry mediator,” a receptor expressed by T lymphocytes) can result into eradication of distant metastases (124).

In another preclinical study, evaluating topical imiquimod (a TLR-7 agonist) and local RT in the TSA murine model of BC with cutaneous metastases, both complete regression of treated lesions and improved distant control and survival were observed (125). This was further shown in nude transgenic mice when imiquimod was associated to IL-10 antibodies, possibly suggesting independence from innate immune response (126).

An overview of ongoing clinical studies in the BC setting is presented in Table 2. Most of them combine RT and immunotherapy in the metastatic setting, often in TNBC. On single study is testing permbrolizumab and preoperative RT in the early setting. All of the studies are early phase and test tolerance of the association, with several testing efficacy in terms of local and distant control, in search of an abscopal effect. The preoperative study tests standard treatment delay and TILs increase (127). In the same spirit, a recent study used radiofrequency ablation and/or single dose preoperative ipilimumab in patients with BC who would anyway undergo mastectomy (91). They observed sustained (persisting at 30 days after mastectomy) immunological responses, such as peripheral elevation in Th-1 type cytokines, activated and proliferating T cells, both CD4^+^ and CD8^+^, as suggested by high **I**nducible T-cell **COS**timulator (ICOS)^+^ and Ki-67, respectively, along with T-reg-associated T-eff cells intratumorally (91).

**Table 2:** Summary of ongoing clinical studies associating RT and immunotherapy in the BC setting (Source: clinicaltrials.gov accessed 03.05.2018).

**Study information**	**PI/Institution**	**Chemotherapy**	**Immunotherapy**	**RT**	**Clinical context**	**Dose**	**Study status**
NCT01421017 Phase I/II	S. Adams New York University School of Medicine	Cyclophosphamide	Imiquimod	conventional	Metastatic Skin metastases 1 site irradiated	5 × 6 Gy days 1, 3, 5, 8 and 10	Completed accrual
NCT02303366 BOSTON II pilot study Observational phase I	S. Loi Peter MacCallum Cancer Centre, Australia	None	MK-3475 (anti-PG-1 antibody)	SBRT	Oligometastatic BC	20Gy	Recruiting
NCT02499367 ONIC Adaptive phase II non comparative5 arms	M.Kok Antoni van Leeuwenhoek	Low dose doxorubicin Cyclophosphamide Cis-platin	nivolumab	RT or SBRT	Metastatic TNBC	20 or 3 × 8Gy	Recruiting
NCT02730130 Single arm phase II	C.Barker Memorial Sloan Kettering Cancer Center	None	Pembrolizumab	RT	Metastatic TNBC	5 × 6Gy	Active, not recruiting
NCT02563925 Pilot	S. Modi Memorial Sloan Kettering Cancer Center	None Trastuzumab if indicated	Tremelimumab	Brain RT or SBRT	Brain metastatic BC		Active, not recruiting
NCT02538471 Phase II	S.Formenti Weill Medical College of Cornell University	None concomitantly	LY2157299 (TGFBR1 inhibitor)	Conventional RT to one metastatic lesion	Metastatic BC	3 × 7.5Gy days 1, 3 and 5	Active, not recruiting
NCT03464942 Randomized Phase II	S. Loi Peter MacCallum Cancer Centre, Australia	None	Atezolizumab	SBRT	Metastatic TNBC	20 vs. 3 × 8Gy	Not yet recruited
NCT03051672 Phase II	S.Tolaney Dana-Farber Cancer Institute	None	Pembrolizumab	Palliative conventional RT	Metastatic BC	5 fractions	Recruiting
NCT03366844 Early Phase I	A. Ho Cedars-Sinai Medical Center	None	Pembrolizumab	Preoperative RT boost	High risk HR+HER-2- or TNBC	3 × 8Gy	Recruiting

## Triple Negative BC (TNBC), Radiotherapy and Immunotherapy

Triple negative BC (TNBC) remains, along with HR-/HER2 positive, among the subtypes with the most aggressive biology, associated to an increased risk of locoregional recurrence and distant failure (15). However, in contrary to the HR^−^/HER2^+^ subtype, systemic treatments have not yet been adequately developed for TNBC and it represents par excellence the BC subtype lacking therapeutical targets (16). It represents an ideal target for the combination of RT and immunotherapy, given that this subtype is the most immunogenic among BC subtypes (78) and that the presence of tumor infiltrating lymphocytes (TILs) within the tumors of patients with early invasive TNBC has been associated with improved prognosis (44).

The association of radiotherapy and immunotherapy has gained extensive attention in the last few years (119, 128) and might be of particular interest in the context of TNBC as this subtype is the most immunogenic among BC subtypes (78).

As already specified above, the median percentage of TILs in TNBC patients is 20% (129) and a 10% increase in intratumoral and stromal TILs translates into a 15 and 17% reduction of risk for recurrence or death and 17 and 27% reduction of risk for death, respectively, in data from the BIG-02-98 study (33). Therefore, if any of the investigational treatments increases sTILs' levels in tumors of TNBC patients, this is expected to be beneficial for those patients. A study of preoperative immunostimulatory SBRT associated to immunotherapy (a toll-like receptor agonist) in the setting of TNBC with the objective to increase TILs is currently in preparation in our Institution. A study with the same objective in a population of TNBC or high risk Luminal non-HER-2 positive patients has recently started accrual in the US (Principal Investigator: Alice Ho, Trial number: NCT03366844), combining preoperative RT and pembrolizumab. Hopefully, these studies will prove the principle of the possibility of TILs increase in this population, susceptible to micrometastatic disease opening the road to larger-scale studies testing outcomes in terms of distant control with the association of RT and immunotherapy.

## Conclusions

The clinical relevance of BC heterogeneity when designing studies in the era of introduction of immunotherapy is pivotal. The combination of radiotherapy and immunotherapy is promising and particularly relevant for immunogenic BC subtypes, such as TNBC or HER-2 positive BC. The availability of a valid biomarker, such as TILs, makes studies of association of immunotherapy and RT very appealing in this context. Combinations of established modalities are nowadays tailored to BC heterogeneity and, accordingly, the design of modern studies of BC requires a careful selection of BC subtypes that are likely to benefit from tailored experimental approaches with a strong translational background. Little is known on the intrinsic properties of radiosensitivity within the BC subtypes (15, 33). Insights on this information will optimize the RT-immunotherapy associations and are expected to lead to tailored studies in selected populations.

The timing of the introduction of immunotherapy with or without immune-stimulatory RT seems to be important, since, the least the tumor burden, the more efficient these treatments are expected to be. Therefore, optimal RT-immunotherapy studies should ideally be designed in the early or oligometastatic setting.

The optimum sequence of RT and immunotherapy remains to be found, as is the ideal dose, fractionation and volume of irradiation. The optimum immunotherapeutic agents to combine with RT are also under investigation. The accumulation of data is rapid and hopefully meaningful insights will be available within the next few years.

In our mind, an ideal RT-immunotherapy study in the setting of BC, should take into account the following elements: (a) focus into subtypes with high mutational load, (b) introduce the combination in early disease or early in the oligometastatic setting, with the scope of micrometastasis eradication by a abscopal effect, (c) use, if possible, fractionated irradiation on the primary, unresected tumor or on the most active metastasis, with fractions in the order of 8 Gy, (d) combine RT with TLR- agonists, that enhance dendritic activation and permit enhanced cross-presentation to T-cells (which permits to take advantage of the major immunogenic effect of irradiation, being the liberation of TAAs) or with the combination of check-point inhibitors and TLR-agonists in order to assist activated T-cells to unmask and kill tumor cells and e. evaluate biomarkers, such as TILs or immune gene signatures, to detect early immune activation, which might otherwise be undetectable in the clinical setting (given that the abscopal effect remains hard to reproduce). These strategies might permit to transform not only the role of RT in BC but also, hopefully and most importantly, might transform not only the role of RT in BC but also, hopefully and most importantly, bring a great therapeutic benefit to patients.

## Author Contributions

All authors listed have made a substantial, direct and intellectual contribution to the work, and approved it for publication.

### Conflict of Interest Statement

The authors declare that the research was conducted in the absence of any commercial or financial relationships that could be construed as a potential conflict of interest.
